# A cross-sectional and longitudinal study evaluating brain volumes, RNFL, and cognitive functions in MS patients and healthy controls

**DOI:** 10.1186/s12883-018-1065-9

**Published:** 2018-05-11

**Authors:** Jessica Frau, Giuseppe Fenu, Alessio Signori, Giancarlo Coghe, Lorena Lorefice, Maria Antonietta Barracciu, Vincenzo Sechi, Federico Cabras, Mauro Badas, Maria Giovanna Marrosu, Eleonora Cocco

**Affiliations:** 1 Multiple Sclerosis Center Binaghi Hospital, Department of Medical Sciences and Public Health, University of Cagliari, via Is Guadazzonis 2, 09126 Cagliari, Italy; 2 Department of Health Sciences, Section of Biostatistics, University of Genova, Via Pastore, 1, 16132 Genoa, Italy; 3 Unit of Radiology, Binaghi Hospital, ATS Sardegna, via Is Guadazzonis 2, 09126 Cagliari, Italy

**Keywords:** Multiple sclerosis, Brain volume, Retinal nerve Fiber layer thickness, Cognitive impairments, BICAMS

## Abstract

**Background:**

The principal biomarker of neurodegeneration in multiple sclerosis (MS) is believed to be brain volume, which is associated with cognitive functions and retinal nerve fibre layer (RNFL). A cross-sectional and longitudinal assessment of the relationship between RNFL, cognitive functions and brain volume.

**Methods:**

At baseline, relapsing patients and healthy controls underwent 1.5 T MRI to estimate the normalized volume of brain (NBV), grey (NGV), white (NWV) and peripheral grey (pNGV) matter. Cognitive functions were evaluated by BICAMS, RNFL by Spectral-Domain OCT. Patients were re-evaluated after 12 months.

**Results:**

Cognitive functions, brain volume, and RNFL differed between the group of 66 patients and that of 16 healthy controls. In the MS group, at baseline, an association was found between: p-NGV and symbol-digit (SDMT) (*p* = 0.022); temporal-RNFL and NBV (*p* = 0.007), NWV (*p* = 0.012), NGV (*p* = 0.048), and p-NGV (*p* = 0.021); papillo-macular bundle-RNFL and NBV (*p* = 0.013), NWV (*p* = 0.02), NGV (*p* = 0.049), and p-NGV (*p* = 0.032). Over the observational period, we found a reduction of brain volume (*p* < 0.001), average-RNFL (*p* = 0.001), temporal-RNFL (*p* = 0.006), and papillo-macular bundle-RNFL (*p* = 0.009). No association was found between OCT, MRI, and cognitive changes.

**Conclusions:**

Brain volume, cognitive functions, and RNFL are continuous measures of different neurodegenerative aspects. BICAMS and OCT have low costs and can be easily used in clinical practice to monitor neurodegeneration.

## Background

Multiple Sclerosis (MS) is a chronic disease involving the central nervous system. The clinical course is generally relapsing at onset, and later progressive [1]. Both the inflammatory and neurodegenerative component is present from the early stages [1]. While the first aspect has been well documented and is monitored in clinical practice using conventional magnetic resonance imaging (MRI), the study of neurodegeneration is more difficult [2]. At present, an important biomarker of the neurodegenerative aspect in MS is the evaluation of brain volume by non-conventional techniques of MRI [3]. Indeed, the loss of brain volume appears to be related to the progression of the disease and the permanent accumulation of disability [3, 4]. One of the most important symptoms related to brain atrophy is the deficit of cognitive functions [5]. This is associated with several MRI markers, in particular grey matter pathology, both in terms of focal lesions and volume loss [5]. Cognitive impairment, which has variable severity and has been identified at all stages of the disease and clinical courses, affects between 40 and 70% of MS patients [6]. It appears to be associated with age, disease duration and disability [7]. Many diagnostic tools are able to explore cognitive functions in MS patients [8], although the majority of these are time consuming and require qualified personnel [8]. However, a recently developed neuropsychological assessment named “Brief International Cognitive Assessment for Multiple Sclerosis” (BICAMS) can be administered by non-specifically trained healthcare staff in around 15 min [8].

In the last decades, optic coherence tomography (OCT), a new instrument for evaluating the neurodegenerative aspect of MS, has been widely studied [9, 10]. OCT is able to evaluate the thickness of the retinal nerve fibre layer (RNFL), a defined marker of axonal loss. RNFL measurement could differentiate MS patients from healthy subjects, and it is clearly related to brain atrophy, disease duration, and disability [10–14]. The most involved RNFL region is the temporal quadrant [15]. The great majority of studies exploring RNFL in MS patients have been performed using a ‘time-domain’ OCT (considered the ‘old’ OCT model), which produces less accurate images than the ‘spectral domain’ OCT (SD-OCT), especially in longitudinal studies [16–19].

BICAMS and OCT share three important qualities for MS assessment in a clinical setting: brevity, ease of use, and low cost.

The present study entails a cross-sectional and longitudinal assessment of the use of SD-OCT and BICAMS in the evaluation of the neurodegenerative aspects of MS. These procedures are compared with MRI brain atrophy measurements, which are currently considered the gold standard among neurodegenerative markers in MS patients.

## Methods

MS-relapsing patients consecutively referred to the MS Centre of the University of Cagliari/Binaghi Hospital from March to July 2015 were prospectively included in the study. A group of healthy controls working in the Binaghi hospital was also recruited over the same time period. All the subjects signed an informed consent. The inclusion criteria for MS patients were: 18-65 years of age; relapsing remitting course; no relapses and/or steroids intake in the 30 days before enrolment and follow-up evaluation. All patients started a disease-modifying drug (DMD) prior to enrolment. Psychoactive drugs or substances that might interfere with neuropsychological performance were forbidden for both patients and healthy controls.

The ethics committee of the University of Cagliari approved the study.

At baseline, after written informed consent was given, all the subjects underwent neuropsychological assessment, brain MRI, and OCT. These three evaluations were carried out within a maximum period of three months. The evaluations were repeated for a group of MS patients after 12 months ±3. The changes from baseline and follow-up (difference between follow-up and baseline) were calculated for all of the collected MRI, OCT and BICAMS parameters collected.

Gender and age were collected for the whole cohort. In the MS group, the following clinical data were also recorded from medical records by a neurologist with expertise in MS: age at onset; clinical course; expanded disability status scale score (EDSS) at baseline; all DMD from six months before baseline until the end of the study; presence of relapses and steroid therapy in the 30 days before baseline and before the follow-up evaluation; and optic neuritis in the clinical history with indication of the affected eye. Eyes affected by previous optic neuritis were excluded from the statistical analysis.

### Neuropsychological evaluation

An expert neuropsychologist (G.F.) performed the neuropsychological evaluation in a quiet room using the BICAMS. This assessment was recently developed by an expert consensus group and included: the Symbol Digit Modality Test (SDMT) to evaluate processing speed (or working memory); the California Verbal Learning Test II (CVLT-II) to evaluate verbal learning and memory; and the Brief Visual Memory Test Revised (BVMT-R) to evaluate visual learning and memory [8, 20]. All tests were considered normal or altered according to the authors’ definition (T score equal or inferior to 35). T score estimations were made using available normative values, with corrections for age, gender and education among the Italian population [20]. Patients were categorized as either cognitive impaired (at least one test altered) or cognitive preserved (no tests altered).

The neuropsychologist was blinded to clinical and radiological findings.

### Brain MR acquisition

Brain MRI acquisition and analysis was performed at the Radiology Unit of Binaghi Hospital, Cagliari, Italy. The operator was blinded to the clinical and neuropsychological status of the subjects. The acquisition of brain MRIs was obtained in a single session, using a Siemens Magneton Avanto Scan at 1.5 T. A sagittal survey image was used to identify the anterior and posterior commissures. A dual-echo, turbo spin-echo sequence (repetition time/echo time 1/echo time 2 5 2075/30/90 milliseconds, 256 X 256 matrix, 1 signal average, 250 mm field of view, 50 contiguous 3 mm slices) yielding proton density–weighted and T2-weighted images were acquired in the transverse plane parallel to the line connecting the anterior and posterior commissures. Transverse T1-weighted (T1W) images (repetition time 35 milliseconds; echo time 10 milliseconds; 256 X 256 matrix; 1 signal average; 2,503,250 mm field of view) were acquired, yielding images of 176 contiguous 1 mm-thick slices, oriented to match the proton density of the T2-weighted image.

Brain parenchyma volumes were measured on T1W gradient echo images by using the cross-sectional version of the SIENA (*structural image evaluation using normalization of atrophy*) software, named SIENAX (part of FSL 4.0: http://www.fmrib.ox.ac.uk/fsl/), a previously described method to estimate global brain volume normalized for head size [21]. MRI analysis allowed for Normalized Brain Volume (NBV), Normalized Grey Matter Volume (NGV), peripheral NGV (p-NGV), and Normalized White Matter Volume (NWV) to be obtained. Lesion refilling was performed as described previously [22].

Longitudinal evaluation of the percentage of brain volume change (PBVC) was performed using SIENA software only in patients not showing new T2 and/or gadolinium enhancing lesions during follow-up. To evaluate annualized PBVC (a-PBVC), the following formula was used: PBVC/months from baseline to follow-up * 12.

### Optical coherence tomography

OCT evaluations were performed using a Spectralis SD-OCT (Heidelberg Engineering; Heidelberg, Germania). The machine is able to record ocular movements via a confocal scanning laser ophthalmoscope (TrueTrac ®; Heidelberg Engineering, Heidelberg, Germany). TrueTrac have adapted the software to ocular movements, allowing a correct examination. Eyes that were blind due to reasons other than MS were excluded. The RNFL examination was performed by a ring scan centred on the optic nerve head. The follow-up study was made using the automatic rescan mode. The thickness of global RNFL, the temporal sector (TEMP) and the papillo-macular bundle sector (PMB) were calculated using the machine’s software, and an evaluation of subclinical previous optic neurotis was performed [23]. After excluding previous clinical and subclinical ON, the minimum value between right and left eye for the same subject was entered for analysis. Two neurologists trained in the use of the Spectralis SD-OCT performed all examinations.

### Sample size and statistical analysis

At an alpha level of 5%, and for a statistical power of 90%, a minimum of 61 patients was needed to observe a significant correlation of at least 0.4 (threshold between a weak and moderate correlation).

Comparisons between MS patients and healthy controls at baseline were made using independent samples Student’s t-test for age, chi-square test for gender, Mann-Whitney for education, and linear regression models for MRI parameters and SDMT. Regression models were adjusted for age (MRI parameters) and education (SDMT).

Partial correlation coefficients were estimated to assess the cross-sectional correlations at baseline between cognitive functions, RNFL and MRI characteristics. These correlations were adjusted for age, gender, EDSS (MRI and RNFL), and education.

A multivariable model for each MRI volume (considered as dependent variable) was performed to assess the impact, quantified by R^2^ value, of each RNFL and cognitive functions characteristics on MRI parameters. Only variables for which univariable analysis showed a *p* value < 0.10 were considered for the multivariable model, and a stepwise approach was adopted to select those included in the model.

One-year changes in MRI, cognitive functions and RNFL were assessed using paired samples Student’s *t*-test.

To assess the correlations between the longitudinal changes in cognitive functions, RNFL and MRI characteristics, partial correlation coefficients were estimated using delta changes between baseline and one year. Correlations between baseline and longitudinal changes were also assessed. These were adjusted for age, gender, EDSS (MRI and RNFL) and education.

Stata (v.14; StataCorp.) was used for the computation of results.

## Results

We included 66 MS subjects (female: 48; 72.7%) and 16 healthy controls (female: 9; 56.25%). Mean age at baseline was 43.4 years (SD: 12) in patients, and 46.8 years (SD: 9) in healthy controls. Mean education was 11.6 years (SD: 4.16) in patients, and 14.3 years (SD: 4.19) in healthy subjects. In the MS group, the mean duration of the disease was 10.8 years and the median EDSS was 2 (range: 0 - 7.5). Ten patients were not included in the OCT study due to poor compliance. In 4 other patients, the OCT evaluation was made only in 1 eye, as the other eye was blind for reasons other than MS.

Baseline values of NBV, NWV, NGV, p-NGV, SDMT, CVLT-II, BVMT-R, average-RNFL, TEMP-RNFL, and PMB-RNFL are reported in Table 1.

**Table 1:** Clinical and demographic features

	MS (*n* = 66)	HC (*n* = 16)	*p*-value
Age, mean (SD)	43.4 (12)	46.8 (9)	0.32
Gender, n(%)			0.21
Females	48 (72.7)	9 (56.3)	
Males	18 (27.3)	7 (43.7)	
Education (years), median (range)	13 (5-21)	13 (8-19)	0.096
Disease duration, mean; median (range)	10.8; 8.5 (0-34)		
EDSS, median (range)	2 (0-7.5)		
NBV, mean (SD)	1473.5 (81.9)	1519.6 (38.4)	0.006^^^
NWV, mean (SD)	694 (39.9)	728.1 (19.1)	0.003^^^
NGV, mean (SD)	777.7 (72)	791.5 (33.1)	0.14^^^
p-NGV, mean (SD)	609.6 (52.1)	616.2 (28.7)	0.11^^^
SDMT, mean (SD)	45.1 (12.3)	53.3 (8.4)	0.037*
CVLT-II, mean (SD)	41.6 (10.3)	–	
BVMT-R, mean (SD)	47.6 (10.8)	–	
RNFL, mean (SD)	93.8 (10.7)	101.8 (9.9)	0.014
TEMP-RNFL, mean (SD)	63.3 (11)	–	
PMB-RNFL, mean (SD)	49.6 (9.4)	–	

^^^ - linear regression model adjusted for Age; *linear regression model adjusted for education

MRI measures (NBV, NWV, NGV, p-NGV) are expressed in ml

Cognitive measures (SDMT, CVLT-II, BVMT-R) are expressed in T score

The comparison between healthy controls and patients at baseline showed higher NBV (*p* = 0.006), higher NWV (*p* = 0.003), and higher RNFL thickness (*p* = 0.014) in the first group. The healthy subjects group also demonstrated better performance at SDMT (*p* = 0.037). No significant differences were found in NGV and p-NGV.

### Cross sectional analysis in MS patients at baseline

**Fig. 1:**
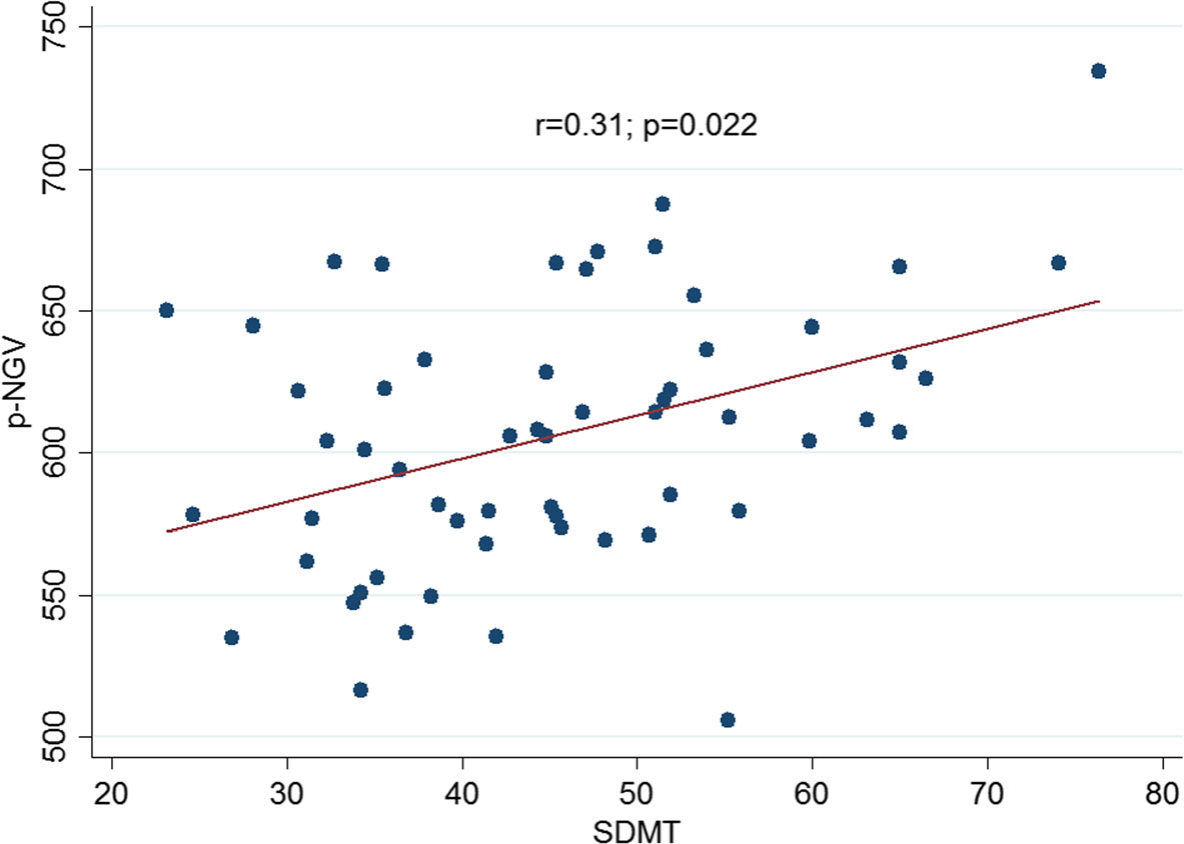
Correlation between SDMT and p-NGV

**Fig. 2:**
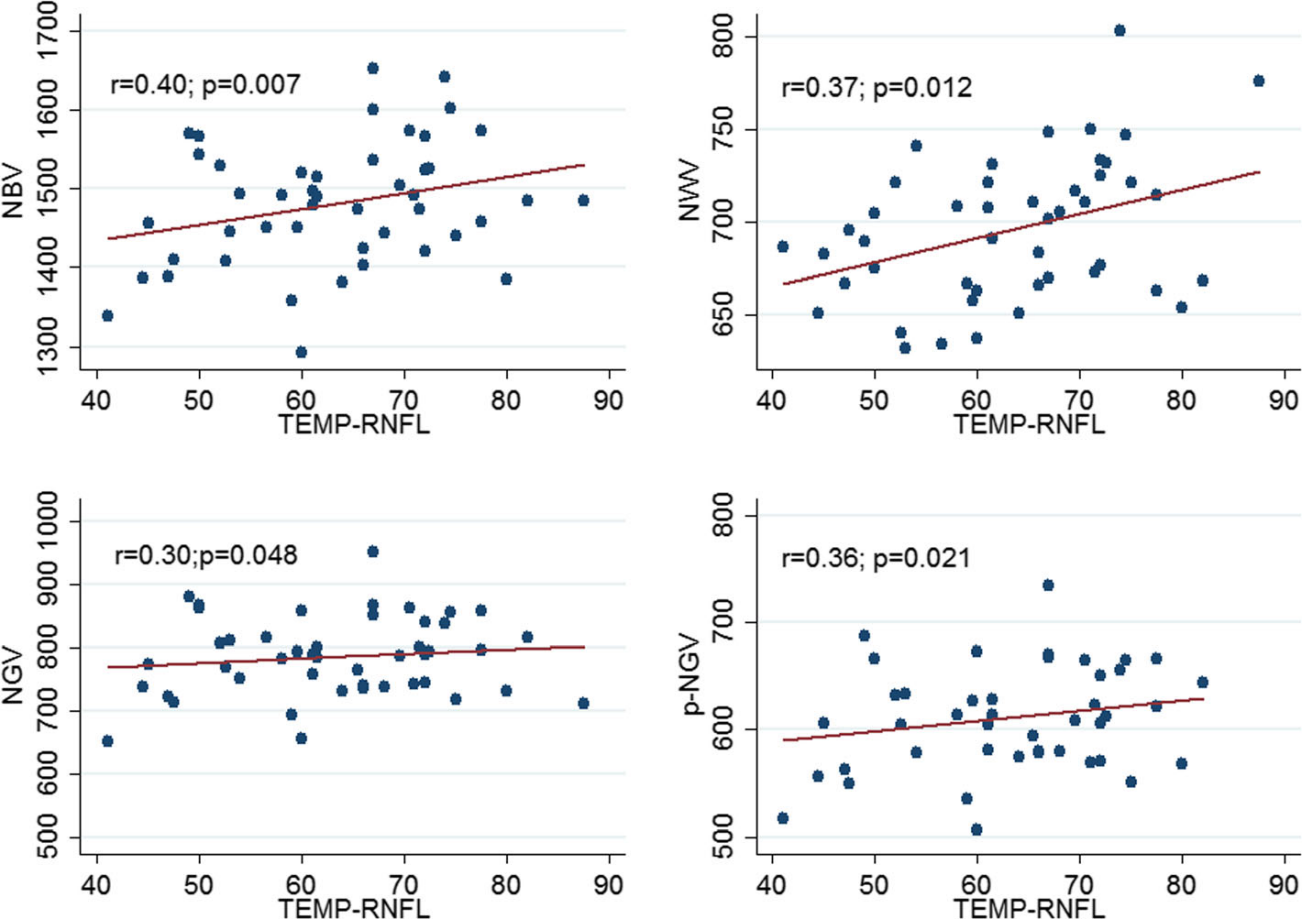
Correlation between TEMP-RNFL and the following brain volume measures: NGV, NBV, NWV, and p-NGV

In Table 2 were reported all baseline correlations. A significant correlation between: p-NGV and SDMT (*p* = 0.022); NGV and SDMT (*p* = 0.013); NGV and BVMT (*p* = 0.048); TEMP-RNFL and NBV (*p* = 0.007); TEMP-RNFL and NWV (*p* = 0.012); TEMP-RNFL and NGV (*p* = 0.048); TEMP-RNFL and p-NGV (*p* = 0.021); PMB-RNFL and NBV (p = 0.013); PMB-RNFL and NWV (*p* = 0.02); PMB-RNFL and NGV (*p* = 0.049); PMB-RNFL and p-NGV (*p* = 0.032). The correlations between SDMT and p-NGV are shown in Fig. 1, while the correlations between TEMP-RNFL and MRI parameters in Fig. 2.

**Table 2:** Partial correlations among OCT, cognitive and MRI parameters

Neurodegenerative assessment	Variables	r	*p*-value
SD-OCT			
Average-RNFL	SDMT	0.01	0.96
	CVLT-II	0.20	0.18
	BVMT-R	0.08	0.60
	NBV	0.25	0.095
	NWV	0.16	0.28
	NGV	0.22	0.14
	p-NGV	0.29	0.06
TEMP-RNFL	SDMT	0.01	0.95
	CVLT-II	0.09	0.57
	BVMT-R	0.08	0.58
	NBV	0.40	0.007
	NWV	0.37	0.012
	NGV	0.30	0.048
	p-NGV	0.36	0.021
PMB-RNFL	SDMT	0.044	0.78
	CVLT-II	0.09	0.55
	BVMT-R	0.13	0.38
	NBV	0.37	0.013
	NWV	0.35	0.02
	NGV	0.30	0.049
	p-NGV	0.33	0.032
SDMT	NBV	0.20	0.10
	NWV	−0.07	0.56
	NGV	0.21	0.11
	p-NGV	0.31	0.022
CVLT-II	NBV	0.09	0.48
	NWV	0.06	0.66
	NGV	0.04	0.78
	p-NGV	0.01	0.97
BVMT-R	NBV	0.11	0.38
	NWV	0.05	0.68
	NGV	0.18	0.17
	p-NGV	0.22	0.11

Average-RNFL thickness displayed a correlation with both NBV (*p* = 0.10), and p-NGV (*p* = 0.06) even thought it did not reach the statistical significance. The OCT measures did not correlate with the results of BICAMS tests.

Since p-NGV correlated with both OCT and cognitive performances, a multivariable model was implemented. After selection, SDMT and TEMP-RNFL, together with age and disease duration, were significantly associated with p-NGV (Table 3). The multivariable model increased R^2^ from 38.3% (with age) to 48.5% with the inclusion of SDMT (∆R^2^ of 10.2%), and to 65.2% with the inclusion of TEMP-RNFL (∆R^2^ of 16.7%).

**Table 3:** Effect of cognitive and OCT characteristics on p-NGV at baseline. The increase of R^2^ associated with the inclusion of significant characteristics in the model was reported

	B (95% CI); *p*-value	∆R^2^
Model 1		
SDMT (T-score)	1.20 (0.46-1.95); *p* = 0.002	+ 0.102
Model 2		
SDMT (T-score)	1.20 (0.44-1.95); *p* = 0.003	
TEMP-RNFL	1.26 (0.35-2.16); *p* = 0.008	+ 0.167

Both models include age and disease duration at baseline

### Longitudinal analysis

At follow-up, 26 patients did not repeat the OCT, 30 did not repeat the brain MRI, and 25 did not repeat the neuropsychological evaluation. The longitudinal evaluations of all the three measures were therefore available for 36 patients, while in a further 4 subjects only OCT and BICAMS, but not MRI, were performed at follow-up.

The mean percentage of brain volume change from baseline to follow-up was 0.57 (SD: 0.54, p: < 0.001). All the OCT parameters changed significantly from baseline to follow-up. A mean decrease of 1.3 (SD: 1.7) for average-RNFL (*p* < 0.001); 1.2 (SD: 1.9) for TEMP (*p* = 0.001); and 1.4 (SD: 2.2) for PMB (p = 0.001) was observed. No significant changes were detected in all the BICAMS tests results.

No significant correlation was found between OCT, MRI and cognitive changes.

## Discussion

We found that the evaluation of RNFL thickness by SD-OCT and cognitive functions by BICAMS are able to differentiate the group of MS patients from that of healthy controls, as previously shown by numerous studies [8–11], as well as being independent markers of neurodegeneration. These were compared with brain volume evaluation performed with MRI - the most commonly used instrument for MS follow-up and what is generally considered the gold standard marker for monitoring the degenerative component of the pathology.

Our data confirmed the association between both assessments and brain volume measures [5, 10–14]. In particular, we found that the BICAMS tests correlate with NGV, especially with cortical atrophy, which is notoriously associated with cognitive decline [5]. Moreover, we found a significant correlation between specific OCT parameters (temporal quadrant and PMB thickness) and brain volume measurements. This correlation appears to be stronger with global and white brain volume than grey matter volume. This result is in line with previous studies in which OCT measures were found to be associated with total brain volume and normalized white matter volume [12, 24], or both normalized white and grey matter volumes [11].

An innovative aspect of our work was the combined analysis of brain atrophy, RNFL thickness, and cognitive functions. Indeed, while the association between brain volume and the other two parameters has been clearly assessed previously, and confirmed by our data, there is less evidence of a possible correlation between RNFL thickness and cognitive impairment. Toledo et al. performed a unique study exploring this issue [25]. They found a significant correlation between SDMT and RNFL thickness, both for the average and the temporal quadrant. However, this result was not confirmed by our work, and we did not observe any correlation between RNFL (average, temporal and PMB) and BICAMS tests. It is worth noting that Toledo et al. performed their OCT analysis with a smaller sample and using a less accurate time-domain machine.

The lack of association between RNFL thickness and cognitive impairment, as for their correlation with different brain volume regions (only grey matter for BICAMS tests, both grey and white matter for TEMP-RNFL and PMB-RNFL), leads us to hypothesize that RNFL and cognitive impairment may represent different aspects of neurodegeneration in MS. Indeed, cognitive decline is secondary to axonal loss, dendritic transection and apoptosis [26]. Moreover, as well as the grey matter pathology, cognitive impairment is mainly associated with primitive degeneration present from the initial stages of the disease, as entirely independent degenerative process, and only partially it is secondary to inflammation. [6, 27]. Otherwise, RNFL thickness may equally be associated with both primitive degeneration and degeneration due to an inflammatory component. In particular, the thinning observed by OCT may be due to clinical and subclinical episodes of ON, primary neurodegeneration of the retinal ganglion cells and their axons, or retrograde transsynaptic degeneration of the ganglion cells and their axons due to MS lesions in the posterior visual pathways [28].

Longitudinally, no significant changes in BICAMS tests scores emerged. This was probably due to a well-known practice effect [20].

A reduction was observed in the mean percentage of brain volume, and in all the OCT parameters considered. However, the longitudinal trend of the two measures did not correlate, perhaps due to the fact that they do not reflect the same specific inflammatory and degenerative brain damage in MS pathology. When considering the brain atrophy measure, we also need to take into account the well-known phenomenon of so-called ‘pseudo-atrophy’ [4], which hinders the correct evaluation of the degenerative and inflammatory components. Thus, some patients could have a ‘false’ light reduction of brain volume over the course of one year due to the presence of inflammatory oedema. To note that all the patients were clinically stable between baseline and follow-up, and no active lesions in brain MRI were observed.

The study was also subject to some limitations.

First, follow-up may have been too short to observe significant changes in the degenerative component, which is present since the initial stages of the disease and is characterized by slow progression.

Second, while our cohort of participants is of a comparable size to that of previous cross-sectional studies, it may be too small to provide clear longitudinal results. Moreover, patients were heterogeneous for the duration of disease, DMD, and grade of inflammation at MRI. However, the great majority of them were clinically stable during the observational period, and no optic neuritis was recorded between baseline and follow-up.

## Conclusions

Our study shows that the evaluation of brain volume, cognitive functions, and RNFL thickness are different continuous measures of neurodegeneration. Unconventional MRI, with its significant costs and requirement for qualified personnel, is more difficult to use in all MS settings. BICAMS and OCT, however, which can be performed at low cost after minimal training, may be suitable for use in clinical practice to assess different aspects of neurodegeneration.

## Abbreviations

a-PBVC: Annualize of percentage of brain volume change
BICAMS: Brief international cognitive assessment for multiple sclerosis
BVMT-R: Brief visual memory test revised
CVLT-II: California verbal learning test II
DMD: Disease-modifying drug
EDSS: Expanded disability status scale
MRI: Magnetic resonance imaging
MS: Multiple sclerosis
NBV: Normalized volume of brain
NGV: Normalized volume of grey
NWV: Normalized volume of white
OCT: Optical coherence tomography
PBVC: Percentage of brain volume change
PMB: Papillo-macular bundle
pNGV: Normalized volume of peripheral grey
RNFL: Retinal nerve fibre layer
SD: Spectral domain
SD: Standard deviation
SDMT: Symbol digit modalities test
TEMP: Temporal
